# Exploring climate‐related gut microbiome variation in bumble bees: An experimental and observational perspective

**DOI:** 10.1002/ecy.70066

**Published:** 2025-03-24

**Authors:** Fabienne Maihoff, Lukas Bofinger, Kristof Brenzinger, Alexander Keller, Alice Classen

**Affiliations:** ^1^ Department of Animal Ecology and Tropical Biology Biocenter, University of Würzburg Würzburg Germany; ^2^ Cellular and Organismic Networks, Center for Organismic Adaptation (CORA), Faculty of Biology LMU Munich Planegg‐Martinsried Germany; ^3^ Animal Ecology Group Institute of Ecology, University of Bremen Bremen Germany

**Keywords:** Alps, altitudinal gradient, bee, climate chamber experiment, elevation, gut symbionts, interaction, microbial composition, pollinator, translocation

## Abstract

Rising temperatures negatively affect bumble bee fitness directly through physiological impacts and indirectly by disrupting mutualistic interactions between bees and other organisms, which are crucial in determining species‐specific responses to climate change. Gut microbial symbionts, key regulators of host nutrition and health, may be the Achilles' heel of thermal responses in insects. They not only modulate biotic interactions with plants and pathogens but also exhibit varying thermal sensitivity themselves. Understanding how environmental changes disrupt microbiome communities is a crucial first step to determine potential consequences for host population responses. We analyzed gut bacterial communities of six bumble bee species inhabiting different climatic niches along an elevational gradient in the German Alps using 16S ribosomal DNA amplicon sequencing. We first investigated whether inter‐ and intraspecific differences in gut bacterial communities can be linked to species' elevational niches, which differ in temperature, flower resource composition, and likely pathogen pressure. A reciprocal translocation experiment between distinct climatic regions tested how the gut bacterial communities of *Bombus terrestris* and *Bombus lucorum* change short‐term when exposed to new environments. Finally, we exposed these species to heat and cold wave scenarios within climate chambers to disentangle pure temperature‐driven effects on the microbiome from other environmental effects. Interspecific variation in microbiome composition exceeded intraspecific variation. Species exhibit varying levels of gut microbiome stability, where stability is defined as the within‐group variance: lower stability, indicated by greater within‐group variance, is predominantly observed in species inhabiting higher elevations. Transplanted species showed subtle short‐term gut microbiome adjustments, marked by an increase in Lactobacillaceae upon exposure to warmer regions; however, the gut microbiomes of these bumble bees did not change under laboratory temperature scenarios. We conclude that marked differences in the gut microbiomes of bumble bees could lead to species‐specific responses to environmental change. For example, less stable microbiomes in bumble bees inhabiting higher elevations might indicate an increased sensitivity to pathogens. Short‐term microbiome changes following translocation indicate that species with relatively stable microbiomes, such as *B. lucorum* and *B. terrestris*, can rapidly integrate new bacteria, which could increase their capacity to cope with new environments under climate change.

## INTRODUCTION

Human‐induced climate change poses a serious threat to global insect biodiversity, including the most important pollinators of alpine ecosystems: the bumble bees (Hymenoptera: *Bombus*) (IPCC, 2018; Sánchez‐Bayo & Wyckhuys, 2019; Soroye et al., 2020). The accelerating rise in temperatures can impact bumble bees either directly via their physiology and/or indirectly through the disruption of their intricate ecological interactions (Maebe et al., 2021; Wisz et al., 2013). Loss or gain of interactions is expected to arise when species depart from their usual distribution boundaries and shift to cooler regions, such as higher latitudes or elevations—a phenomenon already observed under current climate change (Kerr et al., 2015; Maihoff et al., 2023; Marshall et al., 2020; Pyke et al., 2016; Rasmont, Franzen, et al., 2015). While more conspicuous interactions between bumble bees and their food resources and parasites have received considerable attention in the context of bumble bee decline and range shifts (Cameron & Sadd, 2020; Miller‐Struttmann et al., 2015, 2022), interactions between bumble bees and their microbiomes are often overlooked despite their potential impacts on bee nutrition and defense against pathogens (Bosmans et al., 2018; Cisarovsky et al., 2012; Hammer, Le, Martin, & Moran, 2021; Zhang et al., 2021). Understanding if and how environmental factors shape the gut microbiome composition in bumble bees is therefore fundamental to elucidating how these microbial communities potentially influence bees' ability to respond to the ecological challenges they face under climate change.

Beneficial gut microorganisms facilitate the digestion of complex food components, such as the breakdown of pollen, and improve the nutritional quality of nutrient‐poor food (Engel & Moran, 2013; Leonhardt et al., 2022). The gut microbiome composition, at the genus or even strain level, can thereby determine the nutrient content that a bee can extract from a particular plant pollen (Lee et al., 2015; Zhang et al., 2021; Zheng et al., 2016). In addition to its contribution to the digestion process, the gut microbiome plays a critical role in disease prevention (Bosmans et al., 2018; Cariveau et al., 2014; Dosch et al., 2021; Guo et al., 2015). Especially, stable core microbiomes in bumble bees—defined as a consistent community of bacteria that remains relatively unchanged across different individuals (small within‐group variance) and is often characterized by the dominance of bacteria such as, for example, *Gilliamella* and *Snodgrassella*— have been shown to provide resistance to pathogen colonization, thereby protecting the bees against infections and even mortality (Bosmans et al., 2018; Cariveau et al., 2014; Dosch et al., 2021). However, it has also been shown that the presence of such core microbiomes is not essential in bees and that the gut microbiome composition can be more variable (Kwong & Moran, 2016; Li et al., 2015; Zhang et al., 2021).

Two primary mechanisms are known to shape the composition of the gut microbiome and might contribute to compositional differences in the microbiome of bumble bees along climatic gradients. On the one hand, bacteria are taken up by bees from food resources or nesting substrates—a primary mechanism of microbiome acquisition in solitary bees, although this pathway is also relevant in social bees (Keller et al., 2021; McFrederick et al., 2012; Voulgari‐Kokota et al., 2019; Weinhold et al., 2024). These bacteria can differ in their thermal sensitivity (Palmer‐Young et al., 2018) and thus in their ability to endure thermal stress while on flowers or other external habitats (Russell & McFrederick, 2022). Thus, the microbial community present on floral resources or nesting substrates might differ along climatic gradients, such as mountain slopes, which could lead to differences in the microbiome of species that inhabit different elevational niches. Furthermore, given that the microbial communities of flowers can differ between plant species (Gaube et al., 2021; Steffan et al., 2024; Vannette, 2020), the flower choice of a bee may also act as a filter for acquired bacteria.

On the other hand, in social bees, microbes are transmitted between individuals of the same colony through social interactions (McFrederick et al., 2012; Su et al., 2021). Such social interactions seem to explain that many, but not all, bumble bee species show similar microbiome compositions among individuals of the same species (Kwong et al., 2017; Li et al., 2015; Zhang et al., 2021). Fixed associations between bumble bees and gut bacteria can lead to the formation of core microbiomes, enabling the establishment or maintenance of stable microbiomes, which may become vital to counter the expected rising burden of parasites under climate change (Piot et al., 2022; Porras et al., 2023). However, rigidly fixed associations may also pose challenges as climate conditions shift and the bacteria's thermal capabilities and functions no longer align with the changing environment. Conversely, less fixed gut microbiome associations, with adjusted bacterial strains for optimal metabolic efficiency, could also enhance bumble bee success in new environments. In this context, functional redundancy within gut microbiomes could also provide resilience, as multiple bacterial taxa with overlapping functions may compensate for those that cannot adjust to new conditions.

In various other insect and ectothermic species, shifts in gut microbial composition can arise even as a response to brief episodes of heat exposure (Jaramillo & Castañeda, 2021; Sepulveda & Moeller, 2020; Wernegreen, 2012). Such an ability to adjust in the short term could play a pivotal role in coping with fluctuating conditions during the lifetime of an individual, including the anticipated increase in heat and cold waves due to climate change (Lhotka et al., 2018; Rigby & Porporato, 2008). Whether bee gut microbiome composition tends to be determined mostly by species‐specific associations with bacterial strains or whether it can vary as a function of environmental conditions—even in the short term—merits further investigation.

This study addresses the significance of the interplay between abiotic environmental conditions and vegetation in shaping bumble bees' gut bacterial community through three approaches. We aim to shed light on how prevailing thermal conditions that affect both the bacteria and the host may contribute to variations in the gut microbiomes of species residing in diverse climatic niches. First, we asked whether the composition and variability of the gut microbiomes of six coexisting alpine bumble bee species reflect an adaptation to their preferred elevational niche. Elevational gradients provide a suitable proxy for studying the effect of temperature and associated environmental change due to their steep temperature gradients (~0.5°C per 100 m of elevation) that act as a strong filter of both plant and insect communities and the interactions between them (Hoiss et al., 2012, 2015; Körner, 2007; Sponsler, Requier, et al., 2022). Our first objective was to determine how gut microbiomes of bees vary as a function of shifting environmental conditions along an elevational gradient at an interspecific level and how the availability of floral resources and floral choice preference might affect the composition of the gut microbiome. Along an elevational gradient, we can observe changes in the microbiome that can arise from both long‐term influences that are established over generations in a certain elevational niche and short‐term effects that occur within the lifespan of a bumble bee. As a second objective, we specifically examined the short‐term effects of environmental change—those occurring within the lifespan of individuals—and assessed the consistency of the response between species in more detail. For this purpose, we studied how intraspecific gut microbiome composition changed in response to environmental change in two sister species, *Bombus lucorum* and *Bombus terrestris*, whose nests were translocated between different climate regions: a warm and dry region in the lowlands and a cold and wet mountainous region. Lastly, to disentangle purely temperature‐driven effects from combined environmental effects (temperature, precipitation, and resulting vegetation change), we created heat wave and cold wave scenarios in a climate chamber experiment and tested their influence on the gut microbiome composition in the sister species.

## MATERIALS AND METHODS

### Experimental design and sample collection

#### Gut microbiome composition and variance of six bumble bee species differing in elevational niches

**FIGURE 1:**
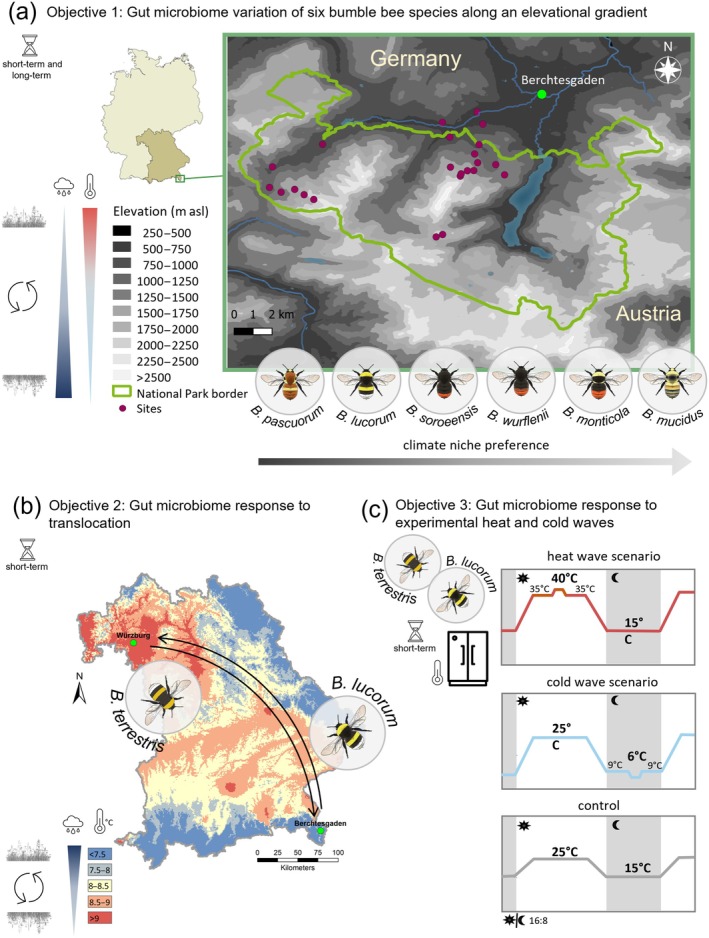
Sampling and experimental design. (a) Study region and sites in Berchtesgaden (Bavaria, Germany), where the gut microbiome of six bumble bee species was related to species' elevational niche preferences. Elevation level is indicated in shades (at 250‐m intervals), with lighter shades signaling increasing elevation. The National Park border is represented with a green line. Points show study sites. Bumble bee species at the bottom of the panel are sorted by their abundance peaks along an elevational gradient. (b) Schematic description of the colony translocation experiment. Queens collected in spring were reared in the lab and then settled with their first offspring in nest boxes. *Bombus lucorum* was caught in the cold and wet region near Berchtesgaden; and *Bombus terrestris* was caught in the warm and dry region near Würzburg. Colonies of both species were established in both regions. The gut microbiome of workers was studied 4–5 weeks after colony establishment in the field. The color code in the map refers to multiannual means of air temperature conditions (30‐year period). (c) Schematic description of the climate chamber experiment. Simultaneously to nest translocations, self‐reared colonies of *B. lucorum* and *B. terrestris* were exposed to either a heat wave scenario (extreme temperature of 40°C), cold wave scenario (extreme temperature of 6°C), or control conditions characterized by the lack of daily extreme temperatures. The gut microbiome was analyzed after 5 weeks. All colonies were exposed to a 16:8 light cycle. Along the elevational gradient both long‐term effects (spanning at least one generation) and short‐term effects (occurring within a worker's lifespan) can play a role. Translocation and climate heat and cold wave scenario only address short‐term effects. Note that changes in precipitation and temperature also affect floral resources, resulting in flower turnover (the circulating arrow marks this expected turnover). Maps were produced in QGIS using data, which was obtained from www.lfu.bayern.de/umweltdaten and www.search.earthdata.nasa.gov and www.dwd.de. Figure created by Fabienne Maihoff using icons of hourglass, cloud, and thermostat from Microsoft. Plant illustrations by Fabienne Maihoff. Bumble bee illustrations by Julia Zetzsche. asl, above sea level.

**FIGURE 2:**
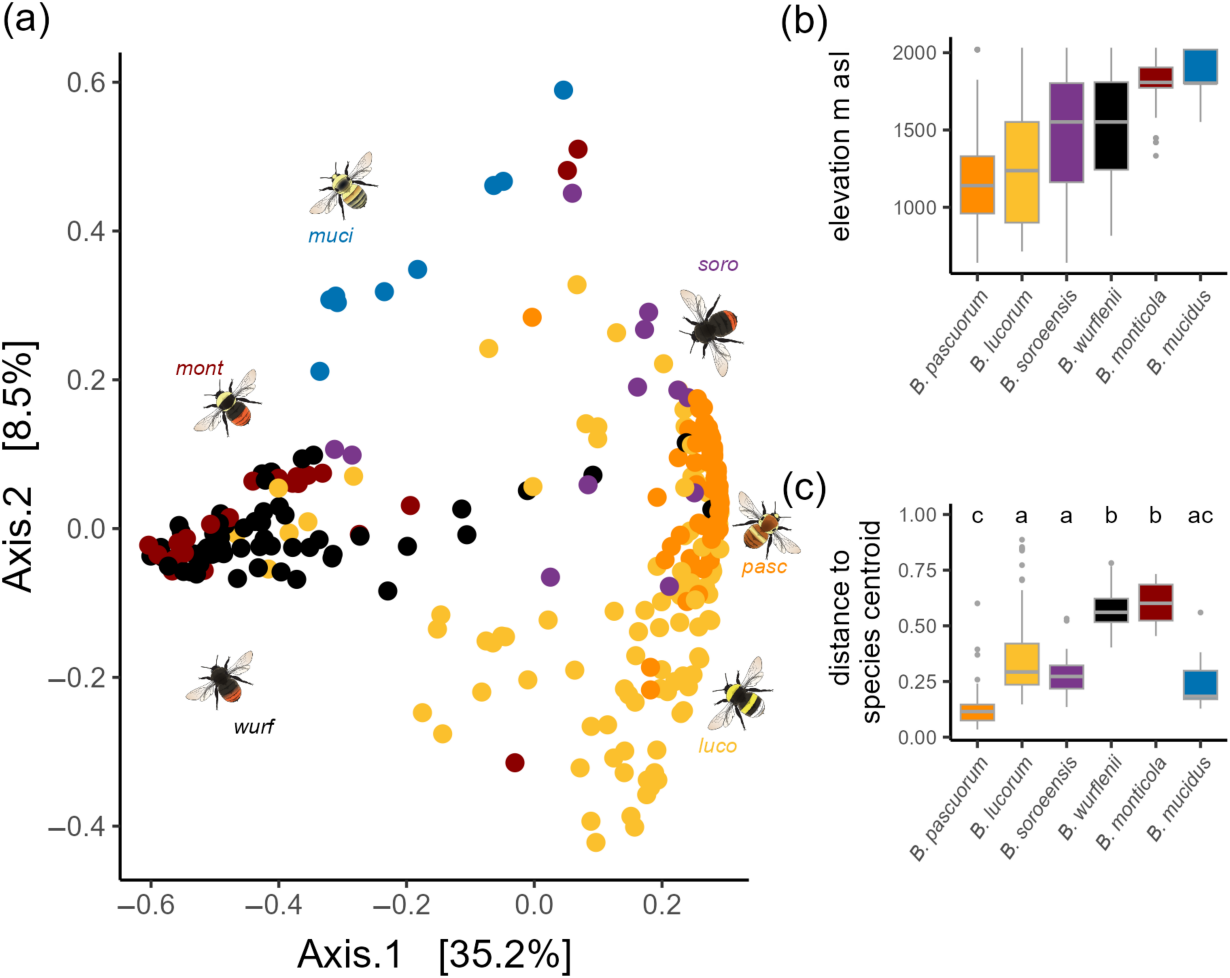
Similarity of the gut microbiome community of six bumble bee species differing in their mean elevational distribution. (a) Principal coordinates analysis based on Bray–Curtis dissimilarities of gut microbiome composition on amplicon sequence variant level. Each dot represents the gut microbiome composition of one individual, colors indicate species (*Bombus pascuorum* = orange; *Bombus lucorum* = yellow; *Bombus soroeensis* = purple; *Bombus wurflenii* = black; *Bombusmonticola* = dark red; *Bombus mucidus* = blue). The closer the dots are to each other, the more similar the microbiome composition. (b) Bumble bee distribution along the elevational gradient recorded during an intensive monitoring conducted between June and September 2019 in the same study region (see Appendix S1: Section S1). (c) Gut variability differences between species. Significant differences (*p* < 0.05) are indicated by letters. Bumble bee illustrations by J. Zetzsche. asl, above sea level.

To test whether differences in the gut microbiome composition and stability between species can be explained by species' elevational niche, bumble bee workers of six species were collected from 23 sites (60 × 60 m plots) covering a gradient from 641 to 2114 m above sea level (asl) within the Berchtesgaden National Park and its close vicinity (47°33′13″ N, 12°55′18″ E) in August 2019 and 2020 (Figure 1a). The National Park is located within the limestone Alps in Southeastern Germany, a region characterized by coniferous forest, alpine meadows, and mountain pastures (Konnert & Siegrist, 2000). We selected species that differed in their elevational distribution. *Bombus mucidus* (Gerstäcker, 1869) and *Bombus monticola* (Smith, 1849) prefer cooler high elevations on average at 1800 m asl. *Bombus soroeensis* (Fabricius, 1777) and *Bombus wurflenii* (Radoszkowski, 1859) mainly occur in mid‐elevations, on average at 1500 m asl. *Bombus pascuorum* (Scopoli, 1763) and *B. lucorum* (Linnaeus, 1761) have their peak abundances at 1200 m asl (monitored in Maihoff et al., 2023, see Figure 2b and further information in Appendix S1: Section S1). Note that the phylogenetic relatedness of the selected species does not correlate with their main elevational distribution (Cameron et al., 2007) (see phylogenetic tree Appendix S1: Section S2).

In total, we collected 290 bumble bee individuals (*B. soroeensis* (*n* = 12), *B. mucidus* (*n* = 9), *B. lucorum* (*n* = 107), *B. monticola* (*n* = 24), *B. wurflenii* (*n* = 62), and *B. pascuorum* (*n* = 76)). Sample size differences between species reflect their natural frequency of occurrences in the study region. High abundances and a wide elevational distribution in *B. pascuorum*, *B. wurflenii*, and *B. lucorum* allowed us to additionally study the intraspecific microbiome patterns of these three species.

All individuals were collected while foraging on flowers to guarantee active interactions with the floral environment. All individuals, except for individuals of *B. lucorum*, were killed and transported on dry ice and stored at −20°C until gut extraction and further laboratory workflow. The gut of 101 *B. lucorum* was directly dissected in the field and stored in 1‐mL DNA/RNA shield (Zymo Research, Eching, Germany) transported on dry ice, and stored at −20°C for further analysis because other body parts were used for a transcriptomic study (Brenzinger et al., 2022). Six individuals of *B. lucorum* were not dissected in the field but were treated equally to the other specimens to check for an effect of methodological differences on microbiome results. Permutational multivariate ANOVA (PERMANOVA) revealed no effect of methodological differences on gut microbiome composition (*F* = 1.60, *R* ^2^ = 0.02, *p* = 0.14).

Species that could not reliably be identified in the field due to morphological similarities were confirmed by DNA barcoding. For this purpose, one leg per individual was used for DNA extraction. DNA extractions were performed using the Qiagen DNeasy Blood & Tissue kit, following the manufacturer's instructions. DNA barcoding was based on the cytochrome oxidase CO1 gene (Hebert et al., 2003, 2004) (AIM—Advanced Identification Methods, Leipzig, Germany) and sequences were annotated through the BLAST (Altschul et al., 1997) and BOLD (The Barcode of Life Data System) databases (Ratnasingham & Hebert, 2007). Of the 108 sampled individuals belonging to the morphologically very similar *Bombus* sensu‐*strictu*‐complex, only one was identified as *B. terrestris*. This did not allow us to study both species along the elevational gradient, and we focused our analysis on *B. lucorum*, excluding the single individual belonging to *B. terrestris*.

#### Gut microbiome response to translocation

To test short‐term gut microbiome composition and stability in response to changing environmental conditions in the closely related sister species *B. lucorum* and *B. terrestris*, we translocated young self‐reared colonies of both species between two distinct climate regions within Bavaria (Germany). Climate regions differ strongly in their mean annual temperature (Figure 1b) and precipitation (Appendix S1: Section S3): the warm and dry region in the Franconian lowlands around Würzburg has an average annual temperature of 9°C and annual precipitation of about 650 mm. The cool and wet region in the Northern Alps around Berchtesgaden has an average annual temperature of 7°C and over 1500 mm of annual precipitation (Figure 1b). In spring, we collected mated queens emerging from diapause in the cold and wet region (*B. lucorum*) and in the warm and dry region in the Franconian lowlands (*B. terrestris*). Queens were reared under constant climatic conditions in a climate chamber (30°C, 60% humidity—following Requier et al., 2020) until at least 11 workers per queen were hatched. When the first workers were observed in the field in their region of origin, we settled the young colonies (queens + workers) in nest boxes. Nest boxes of both species were placed in both climate regions (20 colonies per species). After at least 4 weeks in the field, we collected three specimens per colony and stored them under −20°C until gut extraction. During the translocation period, the warm and dry region was on average 5.4°C warmer and had 223 mm less precipitation than the cold and wet region. In total, we collected 107 individuals (*B. lucorum*: cold/wet region [*n* = 26 out of 9 colonies, 1 colony with only 2 individuals], warm/dry region [*n* = 18 out of 6 colonies]; *B. terrestris*: cold/wet region [*n* = 30 out of 10 colonies], warm/dry region [*n* = 33 out of 10 colonies, 1 colony with 6 individuals]). Species identity of each colony was confirmed by DNA barcoding as described above. Note that predatory ants, early death of the queen, and the presence of wax moths contributed to the loss of colonies.

#### Gut microbiome response to experimental heat and cold waves

To disentangle pure temperature‐driven effects from combined short‐term environmental effects (temperature‐driven differences in floral resources and pathogen changes and precipitation changes), we additionally exposed young self‐reared colonies of *B. lucorum* and *B. terrestris* to heat and cold wave scenarios in a climate chamber experiment and tested their influence on the gut microbiome composition and stability of workers (Figure 1c). Colonies for the climate chamber and the translocation experiment were reared in parallel. After at least 11 workers had hatched, colonies were exposed to three distinct temperature conditions. In the heat wave scenario, temperatures rose to mimic a realistic daily cycle, reaching a maximum of 40°C for 1 h at noon and dropping to 15°C at night. The 40°C refers to the so far measured extreme temperature in the study region on August 7th, 2015, in Kitzingen (DWD, 2017) and is above the temperatures that have been shown to lead to negative behavioral effects in bumble bees (Gérard et al., 2022). In the cold wave scenario, temperature during the day reached 25°C, while the temperature dropped during the night and reached for 1 h a minimum of 6°C. We chose 6°C because bumble bees are known to be inactive at lower temperatures (Oyen & Dillon, 2018). The control scenario was characterized by a daytime temperature maximum of 25°C and a nighttime temperature minimum of 15°C. During the experiment, colonies were fed with pollen (dried deep‐frozen pollen collected from honeybee colonies from the university campus) and sugar water (APIINVERT) ad libitum and experienced a stable 16:8 h light:dark cycle (details see Appendix S1: Section S4). After 5 weeks under treatment conditions, we collected three specimens per colony and stored them in the freezer at −20°C until gut extraction. In total, we collected 54 individuals (*B. lucorum*: heat [*n* = 9], cold [*n* = 12], control [*n* = 12]; *B. terrestris*: heat [*n* = 9], cold [*n* = 12], control [*n* = 9]). Species identity of each colony was confirmed by DNA barcoding as described above.

Since bumble bee workers live between 22 and 69 days (Goldblatt & Fell, 1987; Kelemen et al., 2019; Smeets & Duchateau, 2003), we cannot determine whether the individuals whose microbiomes we studied hatched in the new environments (field or climate chamber, respectively) or if they had already hatched during the colony rearing period. Thus, we consider them exposed to different conditions rather than developed under different climatic conditions.

### Laboratory workflow

After gut dissections in the different experiments (elevational gradient, translocation and climate chamber experiment), guts were placed in 1‐mL DNA/RNA shield (Zymo Research, Eching, Germany) and stored at −20°C. DNA extractions of gut samples were performed using the ZymoBIOMICS DNA Miniprep Kit, following the manufacturer's instructions. Library preparation was performed according to Kozich et al., 2013 including indexing, quality control, normalization, pooling, quantification, and sequencing of the V4 region. This protocol includes dual indexed primers containing the Illumina adapter, index sequence, pad sequence and linker, followed by the gene‐specific primers 515f 5′‐GTGCCAGCMGCCGCGGGTAA‐3′ and 806r 5′‐GGACTACHVGGTWTCTAAT‐3′. Sample‐specific labelling was achieved by assigning a unique forward/reverse index combination to each sample. To minimize amplification biases all PCRs were performed in triplicates and with a proofreading Phusion High‐Fidelity PCR Master Mix with HF Buffer according to manufacturer's instructions (Thermo Fisher Scientific, Waltham, USA). The PCR cycling conditions were as follows: initial denaturation at 95°C for 4 min, 30 cycles of denaturation at 95°C for 40 s, annealing at 55°C for 30 s, and elongation at 72°C for 1 min; followed by a final extension step at 72°C for 5 min. Pooled triplicates per sample were verified for successful amplification using gel electrophoresis in 1.5% agarose gels and stored at 4°C. To ensure uniform sequence coverage, between‐sample normalization was performed using the SequalPrep Normalization Plate Kit (Invitrogen GmbH, Darmstadt, Germany). Before sequencing, we pooled indexed PCR products into plate pools. Library quality and fragment size of plate pools were checked using the High Sensitivity DNA Chip on a 2200 Bioanalyzer (Agilent). DNA concentration was measured using a 1 × dsDNA HS Assay Kit on a Qubit II Fluorometer (Thermo Fisher Scientific). The final library pool was then loaded into a V2 2 × 250 cycle reagent Miseq cartridge according to the manufacturers protocol (Illumina) and sequenced on an Illumina Miseq platform (Department of Human Genetics of the University of Würzburg, Germany). To account for low sequence diversity of the 16S rRNA library, an Illumina 5% PhiXv3 control library was added to the sequencing pool.

The sequences were merged, quality filtered, had chimeras removed and denoised into amplicon sequence variants (ASVs) with VSEARCH (Rognes et al., 2016) using the pipeline available in Github (https://github.com/chiras/metabarcoding_pipeline, Leonhardt et al., 2022). Taxonomy assignment was based on the RDP v18 reference database using SINTAX and threshold 0.8 (Edgar, 2016), also with VSEARCH. Individual ASVs of relevance with low taxonomic resolution here were manually checked with BLAST (Altschul et al., 1997) against the NCBI GenBank (Benson et al., 2018). The raw dataset contained 10,505,024 reads and was clustered into 2844 ASVs. Nonmicrobial reads of host organelles like chloroplasts were removed from the dataset. Only samples with more than 1000 reads were used in further analyzes. The retained bumble bee samples had an average number of 18,716 reads per sample. The median number of reads was 7429. The final dataset contained 2501 ASVs. To account for differences in the sequencing throughput between samples we transformed the sequence read counts of each sample into relative read abundances (RRAs). We decided not to include a rarefaction‐based data trimming but apply relative read abundance in addition to laboratory precautions for equal throughput (triplicate PCR, normalization and equimolar pooling of samples). Rarefaction curves are however provided as a diagnostic measure in Appendix S1: Section S5. Including a rarefaction step does not change the results (Maihoff et al., 2025).

### Statistics

All analyzes were performed using the software R version 4.1.3 (R Core Team, 2021). For data analysis and visualization on ASV and genus level, we used the “*phyloseq*” (McMurdie & Holmes, 2013) and “*microVIZ*” package (Barnett et al., 2021). The R script is published alongside the data repository (Maihoff et al., 2025).

#### Gut microbiome composition and variance of six bumble bee species differing in elevational niches

To determine the similarity of gut microbiome composition between species and between individuals of all studied species, we performed principal coordinates analysis (PCoA) as an unconstrained multidimensional scaling method as an alternative to nonmetric multidimensional scaling (NMDS), which had convergence problems. Dissimilarities between microbiome compositions were calculated using Bray–Curtis dissimilarities. We tested gut microbiome composition differences between bumble bee species and elevation by performing PERMANOVA using the packages “*vegan*” (Oksanen et al., 2020) and “*pairwiseAdonis*” (Martinez Arbizu, 2020). Elevation refers to the elevation at which each bee individual was collected. Please note that abiotic factors such as precipitation and temperature measured as annual means or means during the period of sampling are strongly correlated with elevation (Appendix S1: Section S6). Therefore, we decided to exclusively test elevation within our models, as it includes factors beyond temperature and precipitation and avoids errors due to multicollinearity.

As a measure of stability in the gut microbiome, we determined the within‐group variance of the gut microbiome composition on an inter‐ and intraspecific level. The lower the within‐group variance, the more stable the gut microbiome composition (Hammer et al., 2023). A stable microbiome is expected to respond little to environmental change, which should be reflected in comparable microbiome variance along the elevational gradient. The need to adapt to new floral resources or a release from pathogen pressure in a new environment might, however, also dissolve the stability of the microbiome. To test whether species sharing the same elevational niche have a comparable microbiome variance, we tested for interspecific within‐group variance differences. For this, we calculated the distance to the group centroid (here: group = species) and used a Dunn's Test as a post hoc test after the Kruskal–Wallis test. To test whether the stability of the gut microbiome composition changes along the elevational gradient within a species (= intraspecific stability), we tested for a categorical effect of the elevational gradient on the intraspecific gut microbiome variance by dividing it into three elevational belts (low: 641–1017 m asl mid 1018–1448 m asl; high 1449–2200 m asl). For each individual per species, we calculated the distance to the group centroid (here: group = elevational belt) and tested differences between elevational belts with generalized linear mixed models using the “*lme4*” and “*glmmTMB*” package (Bates et al., 2015; Brooks et al., 2017). Site was included as a random effect. The test was performed with the “Anova” function from the “*car*” package (Fox & Weisberg, 2019). For pairwise post hoc comparison, we used the “*emmeans*” package (Lenth, 2021).

Since gut bacteria of bees are known to be acquired via food resources, albeit to a lesser extent in social bees (Weinhold et al., 2024) than solitary bees (Voulgari‐Kokota et al., 2019), and food resources change along the elevational gradient (Gómez‐Díaz et al., 2023; Rahbek et al., 2019), we investigated the influence of food resources on the composition of the gut microbiome of bumble bees in more detail. In a first step, we explored the effects of the local (study‐site based) plant community, that is, the plant community to which individuals are exposed during foraging, on the microbiome of bumble bees. Plant communities per site were monitored in 2019. We randomly distributed ten 2 × 2 m subplots within each study site at a minimum distance of 5 m between subplots. In each subplot, we assessed all vascular plant species and estimated their respective flower cover, using the Domin scale (Currall, 1987). For analysis, we used a filtered dataset that only includes insect‐pollinated plants (according to Klotz et al., 2002). We performed a redundancy analysis (RDA) to model the effect of the flower composition on the gut bacteria composition (Legendre & Legendre, 2012). Each individual was assigned to its site‐specific flower composition. In a second step, we considered the floral choice preferences of bumble bees. Floral preferences per species were obtained from Sponsler, Kallnik, et al. (2022), which studied bumble bee floral preferences in the same study region in three consecutive years with respect to floral morphotypes and tongue length. We performed a RDA to model the effect of the floral choice preferences of bumble bees provided by Sponsler, Kallnik, et al. (2022) on the gut bacterial composition.

#### Short‐term gut microbiome response to translocation and climate chamber scenarios

To examine short‐term effects of environmental changes—simulated through worker translocation and climate chamber experiments—on gut microbiome composition, we assessed gut microbiome composition similarity among workers using NMDS and subsequent PERMANOVA.

We quantified the stability of the gut microbiome composition within species by analyzing within‐group variance differences between regions or, respectively, climate scenarios based on similarity distances to group centroids. Abiotic factors such as precipitation and temperature measured as annual means or means during the period of translocation correlated strongly with region (Appendix S1: Section S6). Given this strong correlation, we decided to focus exclusively on the region in our models, as it encompasses additional unrecorded factors beyond temperature and precipitation. The multicollinearity of these factors prevents isolating the different drivers of the response. Thus, analyzing region is a proxy for the combined change of increasing temperature and decreasing precipitation among other factors—a pattern expected under climate change where a greater drought severity alongside temperature increase is predicted in most regions (IPCC, 2018).

Each species was analyzed separately. Stability differences between regions were analyzed with linear mixed‐effects models using the “*lme4*” package (Bates et al., 2015). Colony was included as a random effect, given that the gut microbiome in bumble bees is partly transmitted through the colony (Hammer, Le, & Moran, 2021; Su et al., 2021). The test was performed with the “Anova” function from the “*car*” package (Fox & Weisberg, 2019).

To gain valuable insights into a potential common mechanism underlying gut microbiome composition changes, we determined which ASVs can explain between‐group differences based on the mean decrease of the accuracy index in random forest analysis (Breiman, 2001). The mean decrease accuracy is a metric that evaluates the model's performance by measuring the impact of excluding each ASV on the model's prediction accuracy. A higher value of the index indicates that an ASV is important for predicting the group from which the samples come (warm vs. cold). Random Forest analysis was conducted with the “*randomForest*” package (Liaw & Wiener, 2002). With a Kruskal–Wallis test, we tested for the direction of change (rel. abundance increase or decrease of ASVs).

## RESULTS

### Gut microbiome composition and variance of six bumble bee species differing in elevational niches

**FIGURE 3:**
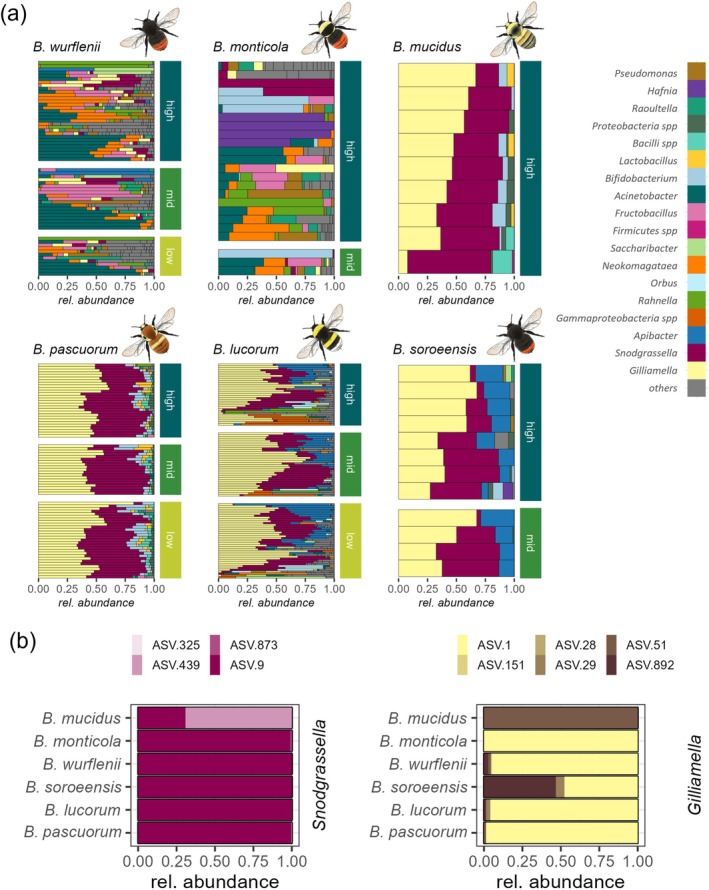
Gut microbiome composition of bumble bees along an elevational gradient. (a) The gut microbiome compositions of bumble bees along the elevational gradient are displayed at the genus level. Colors refer to the 10 most abundant gut bacteria genera (per bumble bee species); gray summarizes all other genera. Rays represent individual gut microbiomes. Individuals were assigned to the elevational belts where they were sampled. Panels are arranged according to the species' respective elevational preferences (from the highest to the lowest: *Bombus mucidus*, *Bombus monticola*, *Bombus wurflenii*, *Bombus soroeensis*, *Bombus lucorum* and *Bombus pascuorum*). (b) The averaged relative abundance of different amplicon sequence variants (ASVs) representing the bacteria genus *Snodgrassella* (left panel) and *Gilliamella* (right panel) per bumble bee species are presented. ASV IDs are given. Bumble bee illustrations by Julia Zetzsche.

Bumble bees gut microbiome composition was mainly explained by species identity (*F* = 35.26, df = 5, *p* < 0.001, *R* ^2^ = 0.38), resulting in species‐specific gut microbiome compositions (Figure 2a, Appendix S1: Section S7, PERMANOVA between all species pairs: *p* = 0.001). Elevation did not predict gut microbiome composition (PERMANOVA: *F* = 1.83, *R* ^2^ = 0.01, *p* = 0.08). The interaction effect between elevation and species was statistically significant but had low explanatory power (*F* = 1.50, *R* ^2^ = 0.02, *p* = 0.034). The intraspecific variability of the gut microbiome composition, measured as the distance to species centroid and reflecting microbiome stability within species, differed among species. Sorted by their elevational niches, bumble bee species that mainly occurred on the lower half of the elevational gradient tended to have a less variant microbiome (*B. pascuorum*, *B. lucorum*, and *B. soroeensis*) than species that mainly occurred on the upper half of the elevational gradient (*B. wurflenii* and *B. monticola*) (Figure 2c). The gut microbiome of the high alpine *B. mucidus* did not fit this trend (Figure 2c): the variance within the gut microbiome was low and comparable with the variance detected in species from lower elevations. This pattern was not influenced by differences in sample size (Appendix S1: Section S8). Interestingly, PCoA on ASV level revealed a higher similarity of the high alpine *B. mucidus* with high alpine *B. monticola* and mountainous *B. wurflenii* compared with *B. lucorum*, *B. pascuorum*, and *B. soroeensis* (Figure 2a,b)*—*a pattern which is not detectable on bacterial genus level (Figure 3a, Appendix S1: Section S9). On bacterial genus level, *B. mucidus* showed a higher similarity to *B. lucorum*, *B. pascuorum*, and *B. soroeensis*. All four species were characterized by a relatively stable gut microbiome composition dominated by the genera *Snodgrassella* and *Gilliamella* (Figure 3a). Divergent ASVs coding for the same bacteria genus in *B. mucidus* (Figure 3b) drive the dissimilarities on ASV level between *B. mucidus* and the group consisting of *B. lucorum*, *B. pascuorum*, and *B. soroeensis*.

Of the three species *B. lucorum*, *B. pascuorum*, and *B. wurflenii*, which we sampled across the entire gradient, elevation explained only very weakly intraspecific changes in gut microbiome composition in *B. wurflenii* (PERMANOVA: *F* = 2.43, *R* ^2^ = 0.04, *p* = 0.008). The compositional changes with elevation were not consistent: the gut microbial composition of mid‐elevation samples differed with low explanatory power from the gut microbial composition of high‐elevation samples (*R* ^2^ = 0.01, *F* = 2.85, *p* = 0.01) but low‐elevation samples did not differ from mid‐ (*R* ^2^ = 0.04, *F* = 1.27, *p* = 0.62) nor high‐elevation samples (*R* ^2^ = 0.04, *F* = 1.79, *p* = 0.13). The intraspecific gut microbiome composition of *B. lucorum* had a higher variance at high elevations compared with mid‐elevations (glmer: χ^2^ = 10.15; df = 2, *p* = 0.006; high vs. mid: *p* = 0.004) while the gut microbiome composition of *B. wurflenii* (glmer: χ^2^ = 1.19; df = 2, *p* = 0.54) and *B. pascuorum* (glmer: χ^2^ = 3.71; df = 2, *p* = 0.16) showed the same intraspecific variability in all three elevational belts (figure shown in Appendix S1: Section S10).

The plant community changed strongly with elevation along the studied gradient (PERMANOVA: *R* ^2^ = 0.30, *F* = 8.96, *p* = <0.001, NMDS shown in Appendix S1: Section S11). In two out of six bumble bee species, *B. wurflenii* and *B. lucorum*, exposed plant communities correlated with their intraspecific gut microbiome compositions (variance explained [RDA]: *B. lucorum*, adj. *R* ^2^ = 0.06, *p* = 0.034; *B. wurflenii*, adj. *R* ^*2*^ = 0.12, *p* = 0.003). Only 6% of the variation in gut microbiome composition of all bees could be significantly explained by species floral preference as a measure of species' diet (variance explained [RDA]: adj. *R* ^*2*^ = 0.06, *p* = 0.001).

### Gut microbiome response to translocation experiment

**FIGURE 4:**
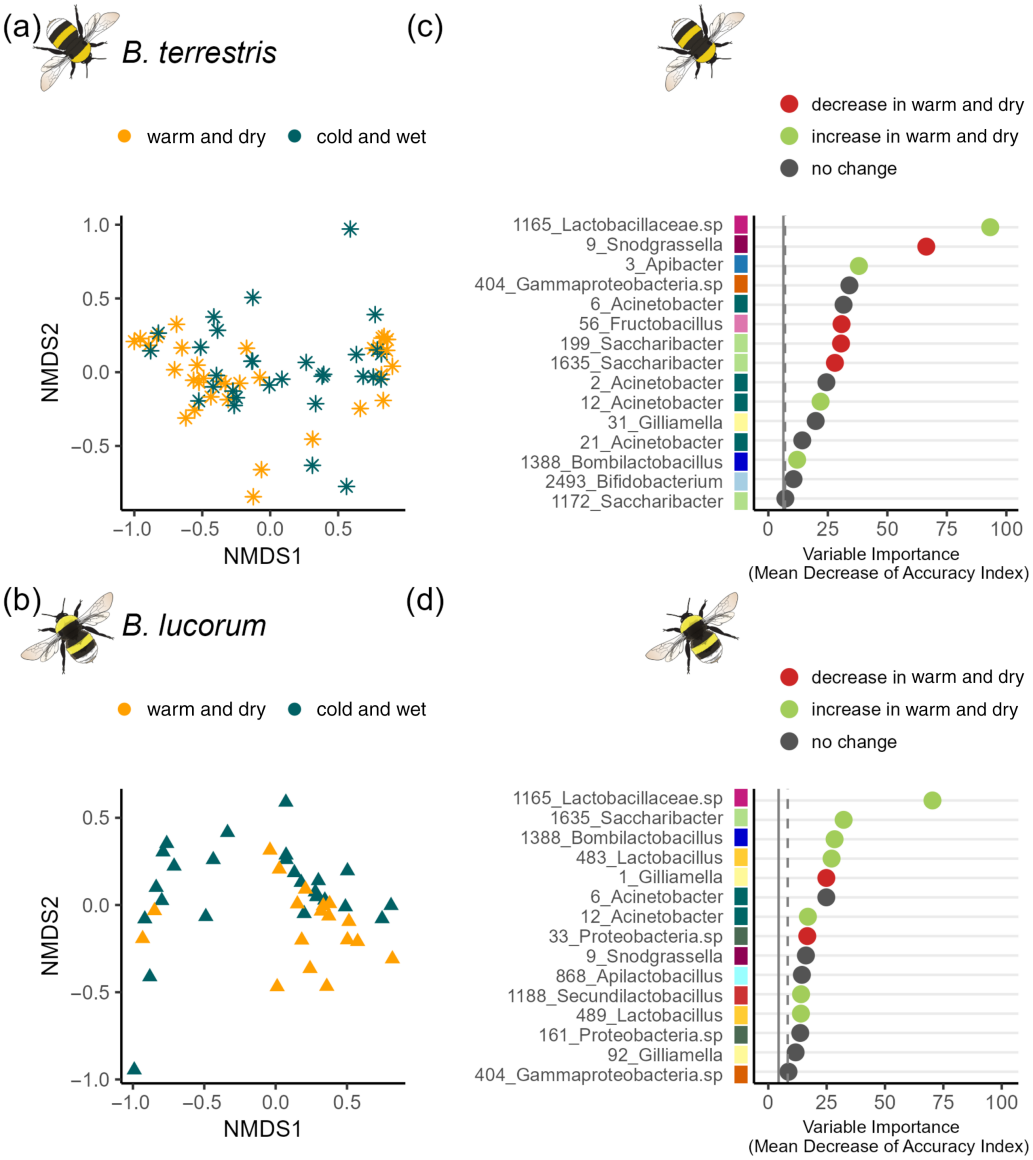
Gut microbiome change in response to bumble bee colony translocation into two different climate regions (warm and dry vs. cold and wet). Similarity of gut microbiome composition of *Bombus terrestris* (a) and *Bombus lucorum* (b) displayed in a two‐dimensional graph by nonmetric multidimensional scaling (NMDS) based on Bray–Curtis dissimilarities. Dots represent individuals, color indicates the region where colonies of individuals have been established (orange = warm and dry; petrol = cold and wet). The closer the dots, the more similar the gut microbiome composition. *B. lucorum* originated from the cold and wet region near Berchtesgaden; and *B. terrestris* originated from the warm and dry region near Würzburg. The 15 most important amplicon sequence variants (ASVs) in distinguishing samples from the warm and dry region and samples from the cold and wet region based on the mean decrease of the accuracy index in random forest analysis are represented in decreasing order for *B. terrestris* (c) and *B. lucorum* (d). High values of the mean decrease in accuracy indicate more important variables in the random forest classification. ASV IDs consist of a unique number and the respective genus or if not further classifiable phylum/family. ASVs of the same genus are represented by the same color. The change direction of a given ASV is colored based on Kruskal–Wallis test results (red = significant decrease in the warm and dry region; green = significant increase in the warm and dry region, gray = no significant change). The vertical solid line represents the average importance of all ASVs in the random forest classification model. Samples right of the vertical dashed line are at least important in 80% of the samples. Bumble bee illustrations by Julia Zetzsche.

Closely related sister species that prefer different climate niches (*B. lucorum* = cold wet vs. *B. terrestris* = warm dry) (Geue & Thomassen, 2020) differed in gut microbiome composition (PERMANOVA: *R* ^2^ = 0.05, *F* = 5.3, *p* = 0.004) and multivariate dispersion (Permutest: *F* = 4.64; *p* = 0.03) in the translocation experiment. In both species, we found only weak overall changes in the microbiome composition after translocation (PERMANOVA: *B. lucorum*, *R* ^2^ = 0.08, *F* = 3.82, *p* = 0.02 and *B. terrestris*, *R* ^2^ = 0.05, *F* = 3.08, *p* = 0.034) (Figure 4a,b). Both species maintained a stable gut microbiome composition (glmer: χ^2^ = 2.66; df = 1, *p* = 0.10 and glmer: χ^2^ = 1.01; df = 1, *p* = 0.32). Interestingly, individuals exposed to the warm and dry region showed consistently higher abundances of an unknown ASV belonging to the Lactobacillaceae, likely of genus *Lactobacillus*. The higher abundance of this ASV explained most of the differences in microbiome composition between climatic regions in both bumble bee species and represented on average 1% of the overall gut microbiome community (Figure 4c,d, Appendix S1: Section S12 Change of ASV test statistic). The compositional changes in the gut microbiome due to bacteria belonging to the Lactobacillaceae (Figure 4) were especially pronounced in the cold‐adapted *B. lucorum*. Among the top predictors (ASVs most important for distinguishing individuals from different regions), the share of ASVs belonging to this bacterial family was greater in *B. lucorum* than in *B. terrestris* (Figure 4c,d). Furthermore, the significant increase in these ASVs was more pronounced in *B. lucorum* compared with *B. terrestris*. On average, the family of Lactobacillaceae represented 7% of the gut microbiome composition.

### Gut microbiome response to experimental heat and cold waves

**FIGURE 5:**
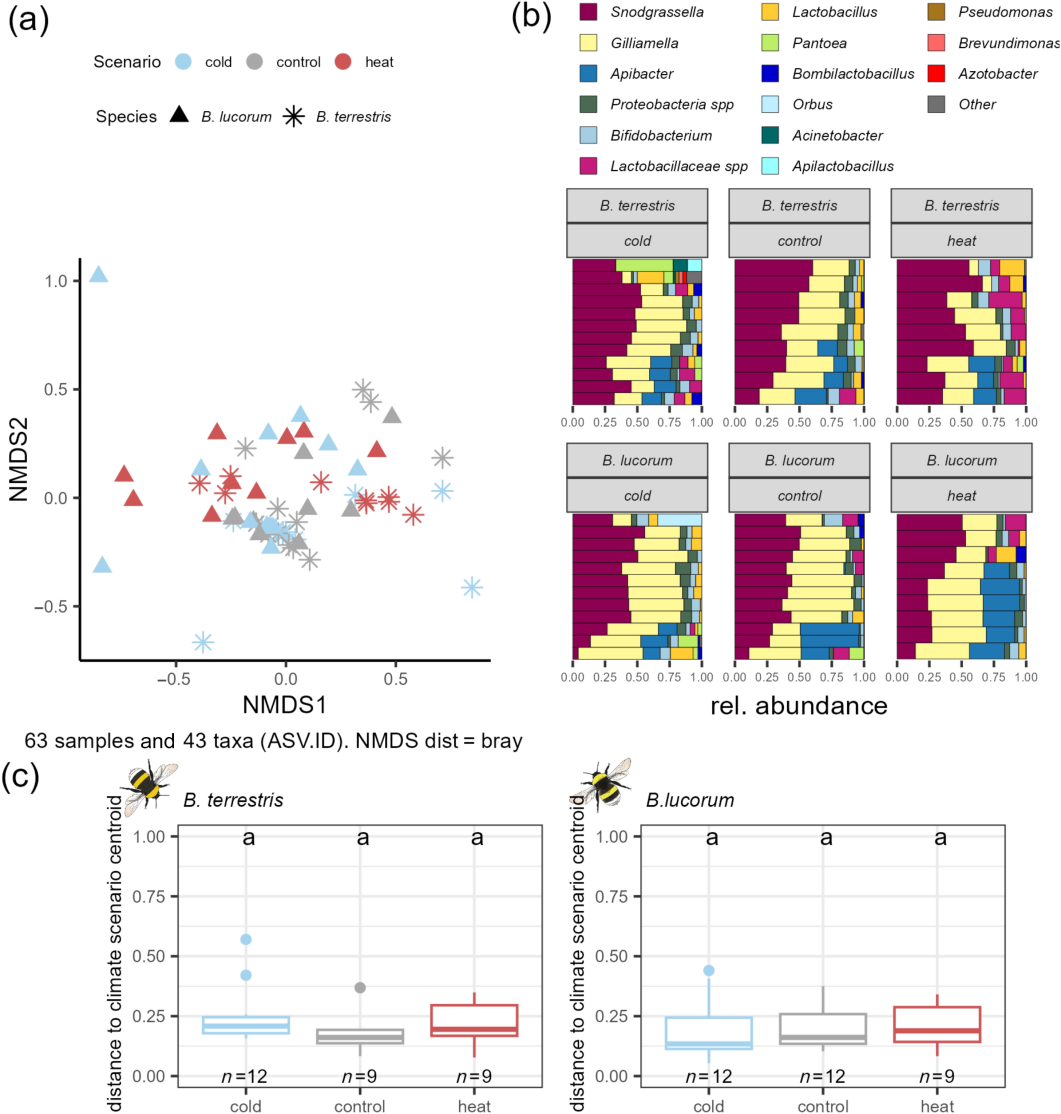
Gut microbiome change in response to heat and cold wave scenarios. (a) Similarity of gut microbiome composition of bumble bees displayed in a two‐dimensional graph by nonmetric multidimensional scaling (NMDS) based on Bray–Curtis distances. Dots represent individuals, dot shapes indicate the respective species (asterisk = *Bombus terrestris*; triangle = *Bombus lucorum*), color indicates the climate scenario individuals were exposed to (blue = cold wave; gray = control; red = heat wave). The closer the dots, the more similar the gut microbiome composition. (b) Stacked bar‐plot representation of microbiota compositions of bumble bees exposed to climate scenarios, with taxonomic features collapsed at the genus level. (c) Stability of the gut microbiome composition in response to climate scenarios based on group‐centroid distances. Bumble bee illustrations by Julia Zetzsche.

Also under controlled laboratory conditions, *B. lucorum* and *B. terrestris* differed in their gut microbial composition (*R* ^2^ = 0.07; *F* = 4.86; *p* = 0.004). In neither species did experimental heat‐ or cold waves change the gut microbiome composition (*R* ^2^ = 0.02; *F* = 0.82; *p* = 0.588) (Figure 5a,b). Heat and cold wave scenarios also had no effect on the stability of gut microbiome composition (glmer: χ^2^ = 0.81; df = 2, *p* = 0.82) (Figure 5c).

## DISCUSSION

We explored patterns of microbial gut composition among bumble bee species exposed to environmental change along elevational gradients, after experimental translocation and under laboratory climate change scenarios (heat and cold waves) to identify biotic and abiotic mechanisms shaping gut microbiomes with respect to bumble bees' responses to environmental change (Hammer, Le, Martin, & Moran, 2021; Sepulveda & Moeller, 2020). In discussing inter‐ and intraspecific changes in the gut microbiome, we address both long‐term alterations and short‐term variations occurring within the worker's lifetime.

### Interspecific differences in the composition and variability of the gut microbiome of six bumble bee species

Interspecific variation of gut microbiome composition exceeded the intraspecific variation of the gut microbiome along the elevational gradient. Interspecific differences in the microbiome composition can arise through various mechanisms, including both selective and nonselective pressures (Koskella & Bergelson, 2020; Yun et al., 2014). In our study, we examine differences between species that inhabit distinct environmental niches, which may offer insights into the potential adaptive role of gut microbiome composition in response to different climatic environments. This perspective was strengthened by selecting species which lack close phylogenetic relationships within the same climatic niche (Cameron et al., 2007, see phylogenetic tree in Appendix S1: Section S2). In this setting, the differences in the interspecific variability of the gut microbiome composition were particularly striking:

Except for *B. mucidus*, species of higher elevation tended to have a greater intraspecific variability, indicating reduced stability in their gut microbiome composition. The absence of consistent abundant associations with *Gilliamella* and *Snodgrassella* in corbiculate bees aligns with recent research questioning the role of these taxa as central microbes for all social corbiculates (Kueneman et al., 2023; Kwong et al., 2017). It is also consistent with the discovery of so‐called enterotypes in bumble bees, as described by Li et al. (2015). They proposed that this absence could result from depletion during overwintering or from replacement by invading environmental bacteria. Overwintering conditions at higher elevations are harsher and potentially more disruptive, with bumble bee queens more likely experiencing temperatures near internal ice formation (Keaveny et al., 2022). The presence of cold‐tolerant *Hafnia* in *B. wurflenii* and *B. monticola* may represent such invading pathogenic bacteria (Wang et al., 2019). The absence of consistent abundant associations with *Gilliamella* and *Snodgrassella* may also indicate that these species already face stronger immune challenges than others (Tian & Moran, 2016), possibly due to temperature constraints that facilitate pathogenic colonization. The microbiome of *B. mucidus*, a species restricted to the highest elevation, however, did not show such high intraspecific variation in the gut microbiome. *B. mucidus* prefers to build nests in warmer microhabitats on south‐facing slopes within these high elevations (Rasmont, Roberts, et al., 2015). This preference may minimize the risk of internal ice formation or invading cold‐tolerant pathogenic bacteria, and might allow this species to maintain a consistent *Gilliamella* and *Snodgrassella* abundance. Interestingly, we found that only when analyzed on the ASV level did distinct discrepancies in the identity of these major symbionts relative to the lowland species become visible. Restricted to our small sample size, we can only speculate that this indicates a potential strain‐level adaptation in the microbiome in *B. mucidus*. Specific bacteria strains that might thrive particularly well in cooler environments (Hammer, Le, & Moran, 2021) can additionally play a role here. To clarify this, comparative studies of bacterial growth optima and within‐nest temperature are needed.

We tested whether the change in vegetation along elevation contributes to the interspecific differences in the gut microbiome. However, we found only weak evidence that differences in flower composition along the elevational gradient and/or differences in floral preferences of species contribute to species‐specific gut microbiome differences. Flower preference explained only 6% of the difference in the gut microbiome between species. The high mobility and capacity for vertical flights of bumble bees (Dillon & Dudley, 2014; Redhead et al., 2016) along with the consequent overlap in their elevational ranges and the comparatively high overlap in floral choice within the genus *Bombus* (Goulson, 2010; Laverty & Plowright, 1988) might explain the low explanatory power of resource availability and preferences. The transfer of microbes between plants, pollinators, and even among pollinators themselves appears to occur predominantly without constraints in our studied species (but see *B. mucidus*).

Other species‐specific traits, such as pH and oxygen levels within the gut (Palmer‐Young, Raffel, et al., 2019; Tegtmeier et al., 2016), differences in the nesting architecture, material, or different pollen storage strategies (Goulson, 2010; Hagen & Aichhorn, 2014) might act as additional filters that further contribute to the differences in microbiome compositions, but these factors could not be considered in our study. Considering only six species, we dismiss a strong phylogenetic or phenological explanation for the observed pattern. For example, the gut microbiome of *B. pascuorum* closely resembles that of *B*. *lucorum*, although *B. monticola* is more closely related to *B*. *lucorum*. In addition, differences in phenology, such as the relatively early emergence of queens in *B. lucorum* and *B. monticola* compared with the rather later emergence in *B. pascuorum* and *B. mucidus*, do not correlate with species distribution along the elevational gradient.

Regardless of the ultimate cause shaping interspecific differences in the stability of the bumble bee gut microbiome, species‐specific differencesmay suggest varying responses to climate change. On one hand, it will be important for species that move to mountaintops as a result of climate change to cope with fluctuating and possibly toxic resources there (Adhikari et al., 2022; Wright et al., 2015). A high variability in gut microbiomes, as detected in *B. wurflenii* and *B. monticola*, coupled with a higher proportion of pollen‐derived bacteria, might facilitate this by appropriate pairing of bacteria and pollen for optimal resource use, as similarly seen in solitary bees (Dharampal et al., 2020, 2022). Considering that pathogen pressure is expected to intensify under climate change, especially in high elevations (Altizer et al., 2013; Harvell et al., 2002; Piot et al., 2022; Porras et al., 2023), species like *B. wurflenii* and *B. monticola* might have a lower ability to protect against pathogens and might also get replaced by other, more resistant species in the alpine bumble bee community in the face of climate change.

### Intraspecific gut microbiome response to changing environmental conditions

Within species, environmental effects on the gut microbiome of foragers along the elevational gradient were weak and did not exceed interspecific differences. In *B. wurflenii* and *B*. *lucorum*, gut microbiome composition changes along the elevational gradient were weakly correlated with changes in the food resources, but not consistent with elevation itself. This points toward the idea that small and flexible changes in the microbiome are driven by bumble bees' interaction with food resources rather than by temperature alone. Both, more established differences between colonies living in different resource communities and/or short‐term responses in an individual's life cycle might shape such intraspecific changes along the elevational gradient.

In contrast to the study of intraspecific changes along the elevational gradient, our translocation experiment specifically focused on the short‐term alterations in the gut microbiome of bumble bees following environmental changes. The composition of the gut microbiome varies between the regions in which the colonies were settled. Numerous factors might impact pure short‐term alterations in our translocation experiment. These factors include variables such as humidity levels, pesticide exposure, and the prevalence of specific pathogens or differences in resource community, among others. Regardless of the individual influencing factors, the composition of the gut microbiome of workers translocated to warm and dry climates was predominantly characterized by an increase in Lactobacillaceae (Phylum:Firmicutes) in both species. Our study is consistent with other studies that have attributed a superior role in warm adaptation to the same phylum (Liu et al., 2022) or family of microbial symbionts (Mayr et al., 2021) or also show an increase under warmer conditions (Palmer‐Young, Ngor, et al., 2019). However, contrasting patterns were also reported (Fontaine et al., 2018; Kohl & Yahn, 2016; Sepulveda & Moeller, 2020; Sudhagar et al., 2017) and question an adaptive value.

Lactobacillaceae bacteria are generally heat‐tolerant; however, certain exceptions exhibit restricted growth at lower temperatures (Matejčeková et al., 2016; Niemand & Holzapfel, 1984). We lack information on the thermal optima of bacteria here, but typical optimal growth temperatures of this group range between 27 and 37°C (Praet et al., 2015). Under such conditions, they might become more competitive in the whole microbiome assembly. Detecting a decrease in relative abundance in cold and wet climates may indicate that the responding ASVs represent strains that cannot grow under extended cold conditions. Further, the finding that the increase of important Lactobacillaceae was more pronounced in *B. lucorum* than in *B. terrestris* (Figure 4c,d) matches with different prerequisites of temperature adaptation in these species: *B. terrestris*, originating from the warmer, dryer climate region, may not need to adjust as much as the more cold‐adapted *B. lucorum*. So far, temperature would be a plausible factor shaping the relative abundance of Lactobacillaceae in bee gut microbiomes. However, the most important ASV was present in field samples along the elevational gradient as well as in samples from the climate chamber experiment. The lack of a similar change within the climate chamber experiment may indicate that the responding strains respond to combined environmental change rather than temperature effects. Lactic acid bacteria have varied proposed functions, including probiotic immune enhancement (Lang et al., 2022; Palmer‐Young et al., 2018; Tejerina et al., 2021) and energy balance regulation for improved heat tolerance (Fontaine & Kohl, 2023; Jaramillo & Castañeda, 2021; Vásquez & Olofsson, 2009). We cannot determine which bacterial functions are involved during translocation because we only considered the cumulative effects of environmental changes between regions, affecting both unknown immune challenges and known heat challenges, and even changes in flower resources. Clarifying the functional role may shed light on whether observed changes could increase bumble bees' capacity to handle new environments under climate change, or whether the observed changes were more passively driven by the presence of bacteria in the environment.

It is noteworthy that the role of *Acinetobacter* in the response to short‐term heat stress, as described in *Drosophila* (Jaramillo & Castañeda, 2021) cannot be confirmed for bumble bees in our experiments, despite detecting *Acinetobacter* in their guts. This could indicate that bumble bees did not experience severe thermal stress in the experimental design or were able to compensate well for it. Bumble bees exhibit a special case in the insect kingdom, which can mitigate and compensate effects: as social ectotherms, they exhibit thermoregulation, maintaining optimal nest temperatures through endothermic‐like behavior (Heinrich, 1974; Vogt, 1986). This thermoregulatory ability likely buffers thermal impacts on their gut microbiome—and may explain rather weak intraspecific effects detected in this study. Additionally, bumble bees' social lifestyle where the queen plays a crucial role in colony establishment (Koch & Schmid‐Hempel, 2011; Su et al., 2021) facilitates the transmission and potential recovery of essential gut microbes following disturbances, helping to maintain the gut microbiome composition across an individual's lifespan (Diouf et al., 2018; Zhukova et al., 2017). This may suggest that our laboratory‐established colonies could also have a stable microbiome that masks immediate environmental effects. Studying bumble bees over multiple generations could thus reveal more pronounced effects of environmental changes.

## CONCLUSION

Our study highlights the variability of the gut microbiome within and among six *Bombus* species along an elevational gradient. Each species exhibited distinct microbiomes, suggesting that long‐term associations and successful social transmission shape their gut microbiomes. The multifaceted functions of the gut microbiome make it challenging to disentangle changes attributable solely to abiotic factors from those influenced by concurrent shifts in biotic interactions. However, due to interspecific variation in their microbiome composition, species may respond differently to the indirect effects of climate change, including increased pathogen levels or shifts in resource communities. Small intraspecific changes indicate the potential for bumble bees to adjust to environmental change despite fixed bacterial associations that may have developed over generations. Overall, understanding these dynamics is crucial as climate change continues to alter habitats and influence both bumble bee microbiomes and their hosts' health and survival.

## CONFLICT OF INTEREST STATEMENT

The authors declare no conflicts of interest.

## Supporting information


Appendix S1.

## Data Availability

Sequencing data of bacterial composition based on 16S metabarcoding and the raw sequence FASTA file for bumble bee identification are available in the National Center for Biotechnology Information (NCBI) Sequence Read Archive under accession number PRJNA1036403 at https://www.ncbi.nlm.nih.gov/bioproject/PRJNA1036403. Data and R code (Maihoff et al., 2025) are available in Figshare at https://doi.org/10.6084/m9.figshare.24533677.v1.
