# Supplementary material for: Exploring climate‐related gut microbiome variation in bumble bees: An experimental and observational perspective
Source: Ecology. 2025 Mar 24;106(3):e70066. doi: 10.1002/ecy.70066 (PMC11933737; doi:10.1002/ecy.70066)
Supplement: Supplementary file 1 — Appendix S1. [file ECY-106-e70066-s001.pdf]

Appendix S1: Exploring climate-related gut microbiome variation in bumble bees: An experimental and observational perspective

Fabienne Maihoff, Lukas Bofinger, Kristof Brenzinger, Alexander Keller, Alice Classen

Section S1: Species preferred elevational niche

The elevational distribution of the six selected bumble bee species was derived from a previous intense wild bee monitoring undertaken in 2019 (Maihoff et al., 2023) in the same region and on additional sites. The gradient in elevation varied from 641 m to 2032 m above sea level (m.a.s.l.). In 2019, 33 grassland areas (60 x 60 m) were chosen along 5 slopes. Wild bees and honeybees were observed from May to September, spanning the whole foraging season. Monitoring was carried out in standardized 50-minute transect walks, which were repeated six times in lower elevations (<1200 m.a.s.l.) and five times in higher elevations owing to the shorter season. Monitoring took place from 9:30 to 18:00 when the weather was sunny or when temperature at 650 m.a.s.l. was above 17 °C on cloudy days. We caught the insects with sweep nets for subsequent morphological identification to species level. Even though we did not use molecular tools to identify species in this monitoring, we are confident that this data accurately reflects the distribution of *B. lucorum* and is not confounded with morphological very similar sister-species, given that in 2019 we found only one male of *B. terrestris* among 117 analysed samples collected for this study in the alpine study region (Brenzinger et al., 2022).

Section S2: Phylogenetic relationship between studied species

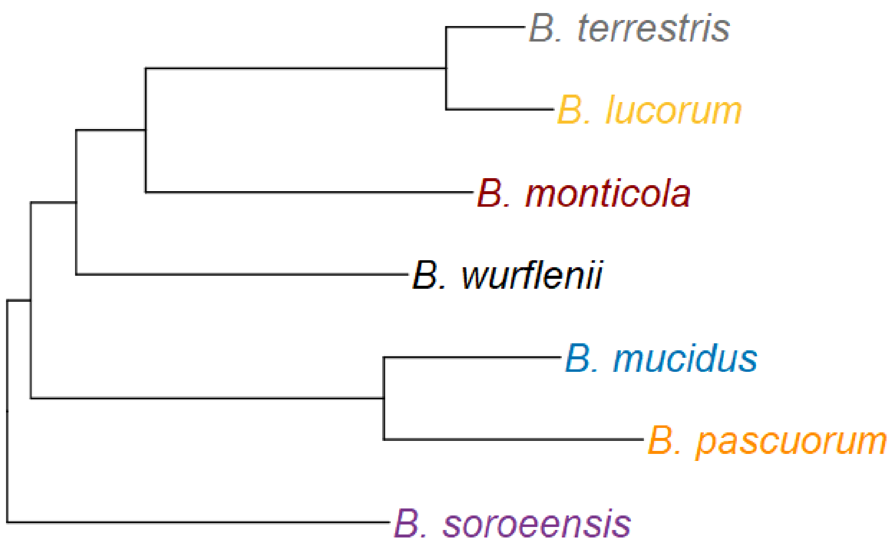

Figure S1. *Phylogenetic Tree of studied species based on data provided from Cameron et al. 2007*

Section S3: Precipitation in study region

Multi-annual means of precipitation (Supplemental Figure 1) and air temperature for the entire region Bavaria are obtained from DWD Climate Data Center (CDC). Multi-annual means refer to a 30 year mean (1981-2010) at a spatial resolution of 1km x 1km.

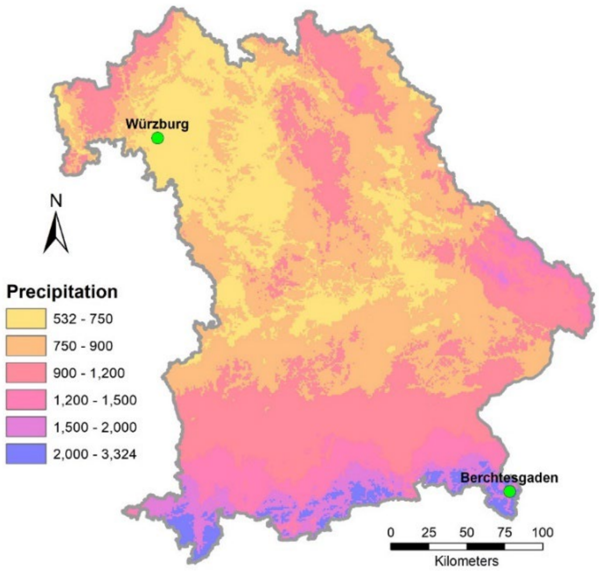

Figure S2. Precipitation characteristics within the translocation experiment. *Climate regions differed substantially in annual precipitation. Multi-annual (30 years) mean precipitations (in mm) are presented.*

Section S4: Hourly conditions during the climate chamber experiment

Table S1. Hourly conditions during the climate chamber experiment. *Given are humidity, light conditions and temperature conditions for the respective treatments (control, heat wave scenario, cold wave scenario)*

time (24h clock)	humidity	light 16:8	temperture (°C) control	temperture (°C) heat wave scenario	temperture (°C) cold wave scenario
00:00 to 01:00	65%	off	15	15	9
01:00 to 02:00	65%	off	15	15	6
02:00 to 03:00	65%	off	15	15	9
03:00 to 04:00	65%	off	15	15	9
04:00 to 05:00	65%	off	15	15	9
05:00 to 06:00	65%	off	17	17	12
06:00 to 07:00	65%	on	17	20	14
07:00 to 08:00	65%	on	20	25	20
08:00 to 09:00	65%	on	20	25	20
09:00 to 10:00	65%	on	23	30	23
10:00 to 11:00	65%	on	23	30	23
11:00 to 12:00	65%	on	25	35	25
12:00 to 13:00	65%	on	25	35	25
13:00 to 14:00	65%	on	25	40	25
14:00 to 15:00	65%	on	25	35	25
15:00 to 16:00	65%	on	25	35	25
16:00 to 17:00	65%	on	25	35	25
17:00 to 18:00	65%	on	23	30	23
18:00 to 19:00	65%	on	23	30	23
19:00 to 20:00	65%	on	20	25	20
20:00 to 21:00	65%	on	20	25	20
21:00 to 22:00	65%	on	17	20	14
22:00 to 23:00	65%	off	17	17	12
23:00 to 24:00	65%	off	15	15	9

Section S5: Rarefaction plot

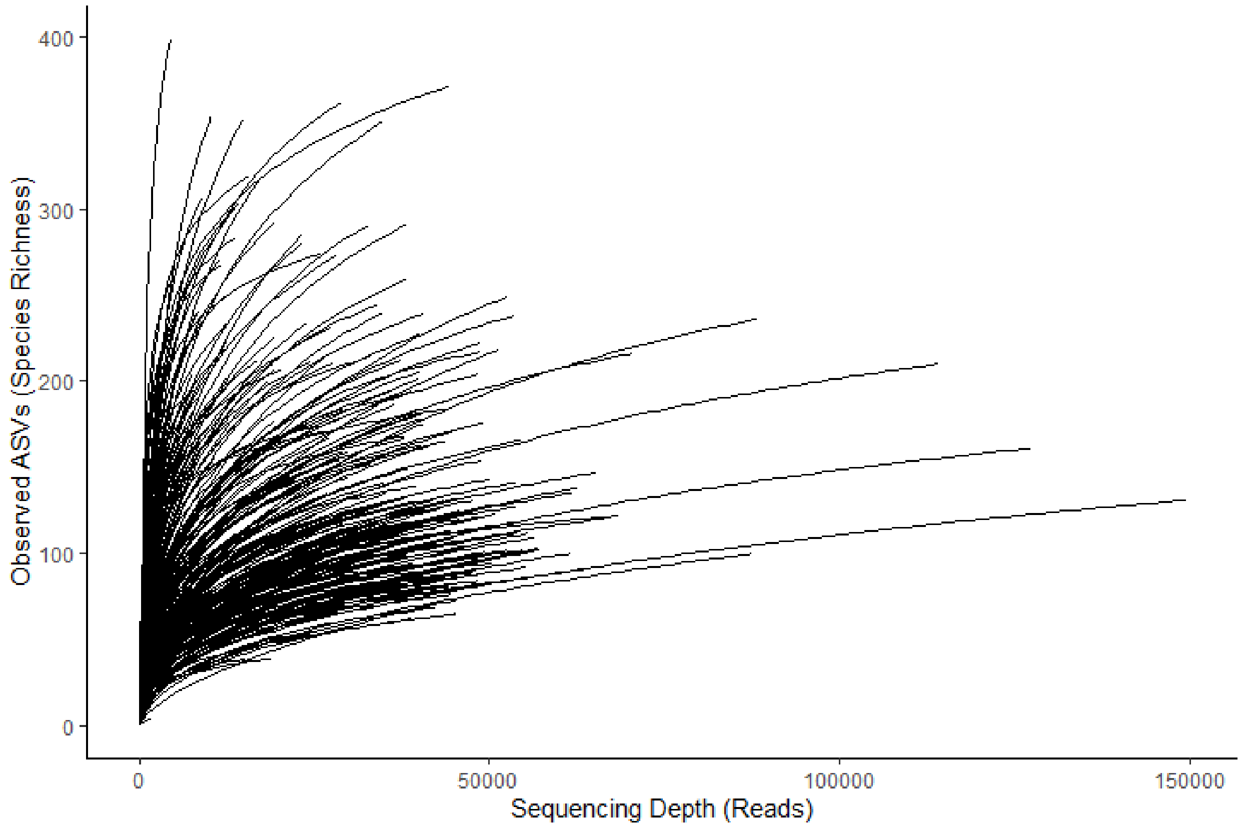

Figure S3. Rarefaction curves showing the number of ASVs as a function of sequencing reads.

Section S6: Multicollinearity of temperature and precipitation within the study design

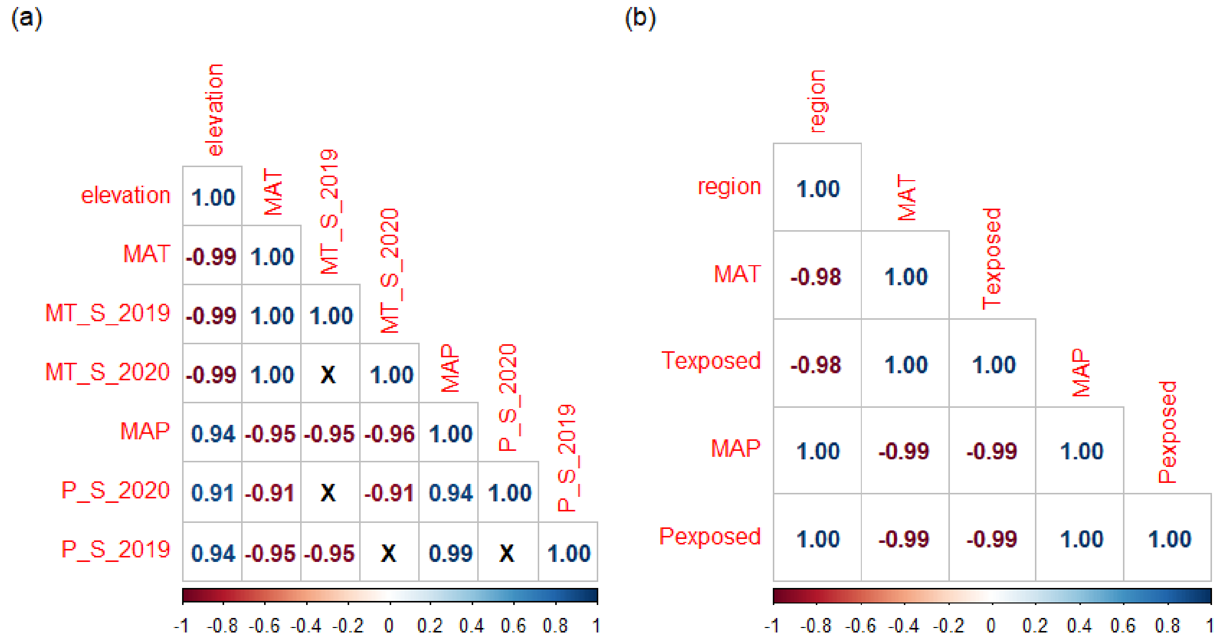

Figure S4. *Multicollinearity along an elevational gradient. Climate factors just as temperature and precipitation strongly correlate with elevation along the elevational gradient (a) and respective region within the translocation experiment (b). Presentet are the correlation coefficients for mean annual temperature (MAT); mean annual precipitation (PAT), mean temperature during the period of sampling along the elevational gradient in 2019 (MT_S_2019) and 2020 (MT_S_2020) , mean precipitation during the period of sampling along the elevational gradient in 2019 (P_S_2019) and 2020 (P_S_2019), the temperature at the location of translocation to which colonies where exposed during translocation (Texposed) and the precipitation at the location of translocation to which colonies where exposed during translocation (Pexposed). Due to this strong multicollinearity, it is impossible to disentangle single effects. Therefore, we decided to only include elevation or respectively region into the model, which describes the combined effect of these measured parameters.*

Section S7: Pairwise Permanova statistic to reveal differences between species

Table S2. Pairwise test (Tukey) for homogeneity of multivariate dispersions and Pairwise Permanova to reveal differences between species. *Within-group variation can be confounded with a between-group variation (Anderson, 2001), therefore, we present the analysis of multivariate homogeneity with the function ´betadisper´ and ´permutest´ from the ´vegan´ package to test whether the species differ in their dispersion, too. Permutations were set on 999.*

**Species comparison**	**Pairwise homogeneity test**				**Pairwise Permanova**		
	**diff**	**lwr**	**upr**	**p adj**	**F-value**	**R2**	**p-value**
B. monticola vs. B. lucorum	0.27	0.17	0.37	**<0.001**	34.95	0.21	**0.001**
B. mucidus vs. B. lucorum	-0.08	-0.23	0.08	0.683	32.65	0.22	**0.001**
B. pascuorum vs. B. lucorum	-0.22	-0.28	-0.15	**<0.001**	23.17	0.11	**0.001**
B. soroeensis vs. B. lucorum	-0.04	-0.18	0.09	0.943	13.36	0.10	**0.001**
B. wurflenii vs. B. lucorum	0.24	0.16	0.31	**<0.001**	56.89	0.25	**0.001**
B. mucidus vs. B. monticola	-0.35	-0.52	-0.17	**<0.001**	10.75	0.26	**0.001**
B. pascuorum vs. B. monticola	-0.49	-0.59	-0.38	**<0.001**	79.77	0.45	**0.001**
B. soroeensis vs. B. monticola	-0.31	-0.47	-0.15	**<0.001**	11.56	0.25	**0.001**
B. wurflenii vs. B. monticola	-0.03	-0.14	0.07	0.949	3.38	0.04	**0.001**
B. pascuorum vs. B. mucidus	-0.14	-0.30	0.02	0.122	156.17	0.65	**0.001**
B. soroeensis vs. B. mucidus	0.04	-0.16	0.23	0.995	30.82	0.62	**0.001**
B. wurflenii vs. B. mucidus	0.32	0.16	0.47	**<0.001**	13.18	0.16	**0.001**
B. soroeensis vs. B. pascuorum	0.17	0.04	0.31	**0.005**	54.70	0.39	**0.001**
B. wurflenii vs. B. pascuorum	0.45	0.38	0.53	**<0.001**	94.36	0.41	**0.001**
B. wurflenii vs. B. soroeensis	0.28	0.14	0.42	**<0.001**	12.84	0.15	**0.001**

Section S8: Sample size effect on the stability of the gut microbiome composition

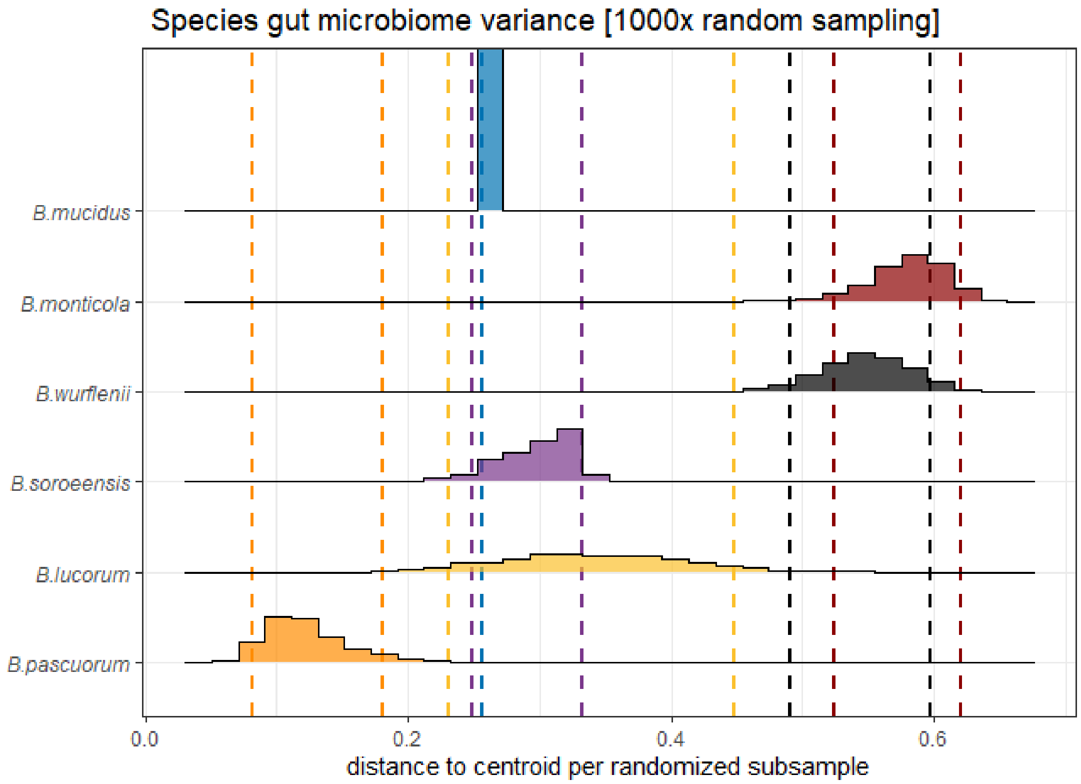

Figure S5. Randomized gut microbiome composition variance per species to test stability over sample size. *To test the effect of sample size differences between species on variance differences, the lowest samples size (here B. mucidus n = 9) was randomly sampled for every other species. Presented are density riches per 1000 random samples. Horizontal dashed lines represent the 75% confidence interval per species. An overlap indicates no significant difference between species. Overall, the results show that irrespectively of the sample size the variance differences between species remain.*

Section S9: Similarity of the gut microbiome community of six bumble bee species based on genus-level of bacteria

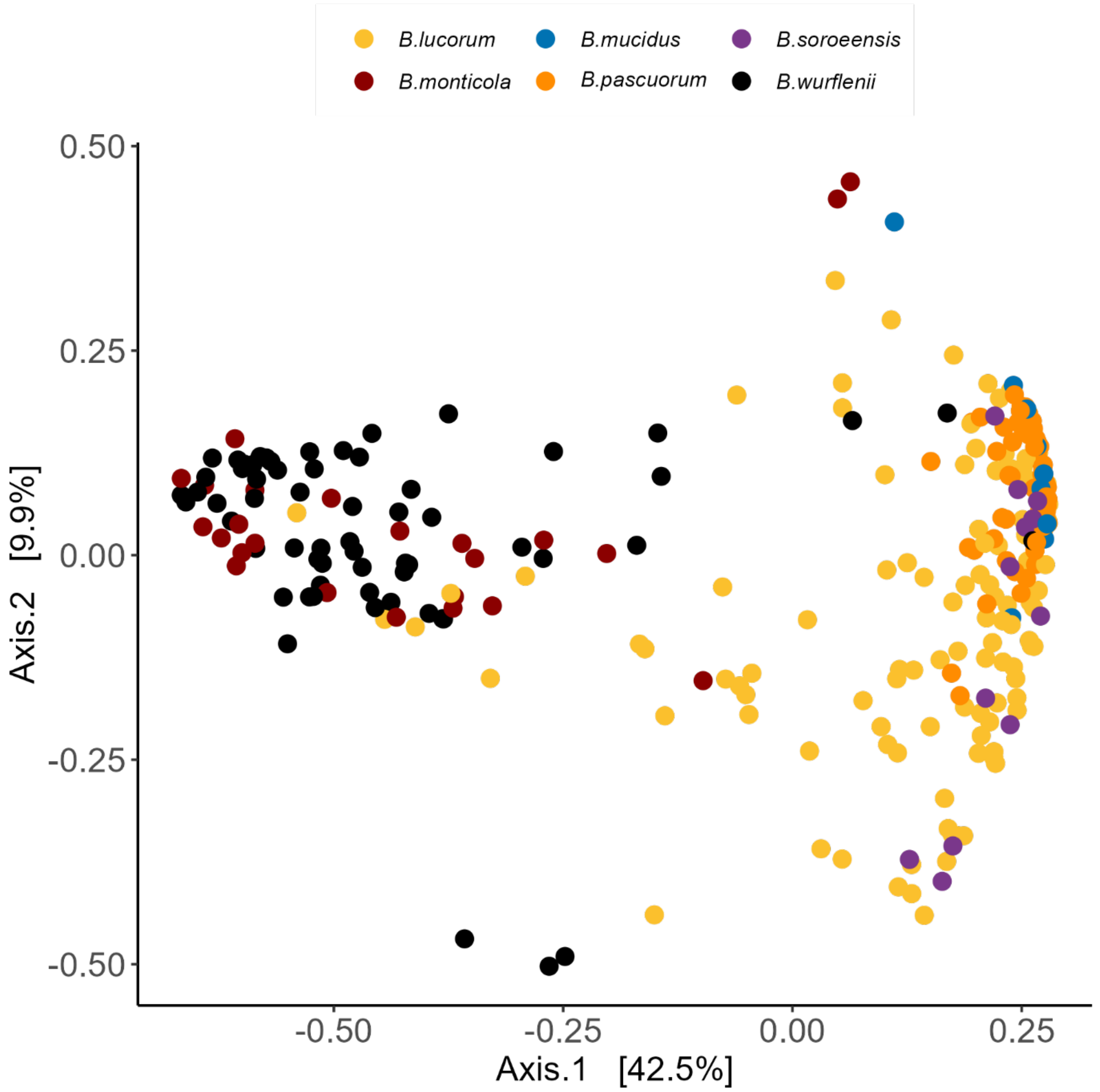

Figure S6. Principal Coordinates Analysis (PCoA) based on bray-curtis distances of gut microbiome composition on genus-level. *ASVs per genus were summarized. Each dot represents one sample, color indicate species (*B. pascuorum = *orange;* B. lucorum = *yellow;* B. soroeensis = *purple;* B. wurflenii = *black;* B. monticola = *dark red;* B. mucidus = *blue). The closer the dot the more similar two samples.*

Section S10: Intraspecific stability of the gut microbiome composition across elevational belts

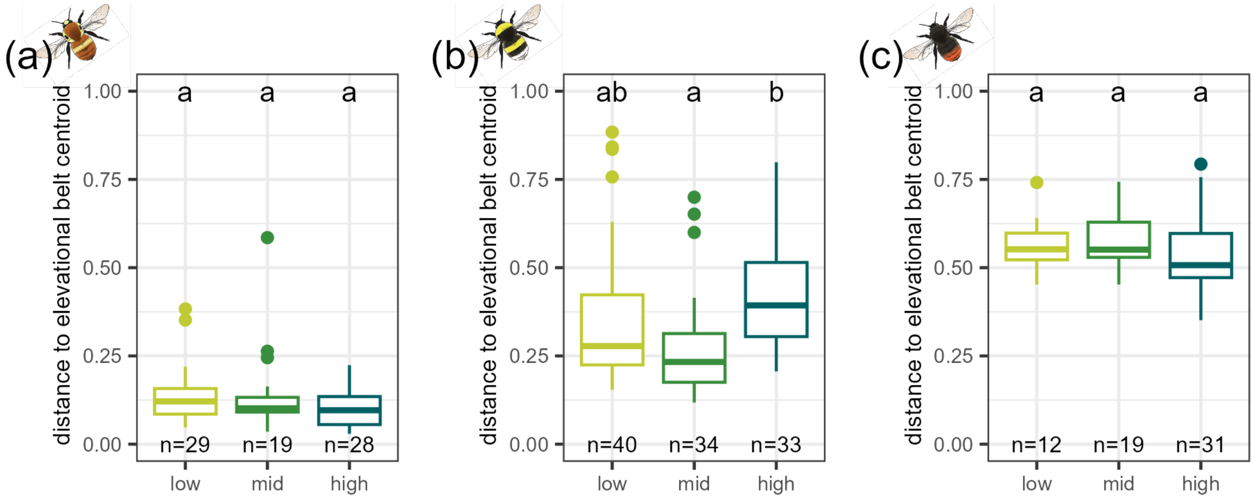

Figure S7. Variance differences along elevational belts *(low: <= 1017 m.a.s.l. mid 1018- 1448 m.a.s.l.; high 1449- 2200 m.a.s.l) in B. pascuorum (a), B. lucorum (b) and B. wurflenii (c). Significant letters and sample sizes are displayed. Bumble bee illustrations by Julia Zetzsche.*

Section S11: Plant community composition change along the elevational gradient.

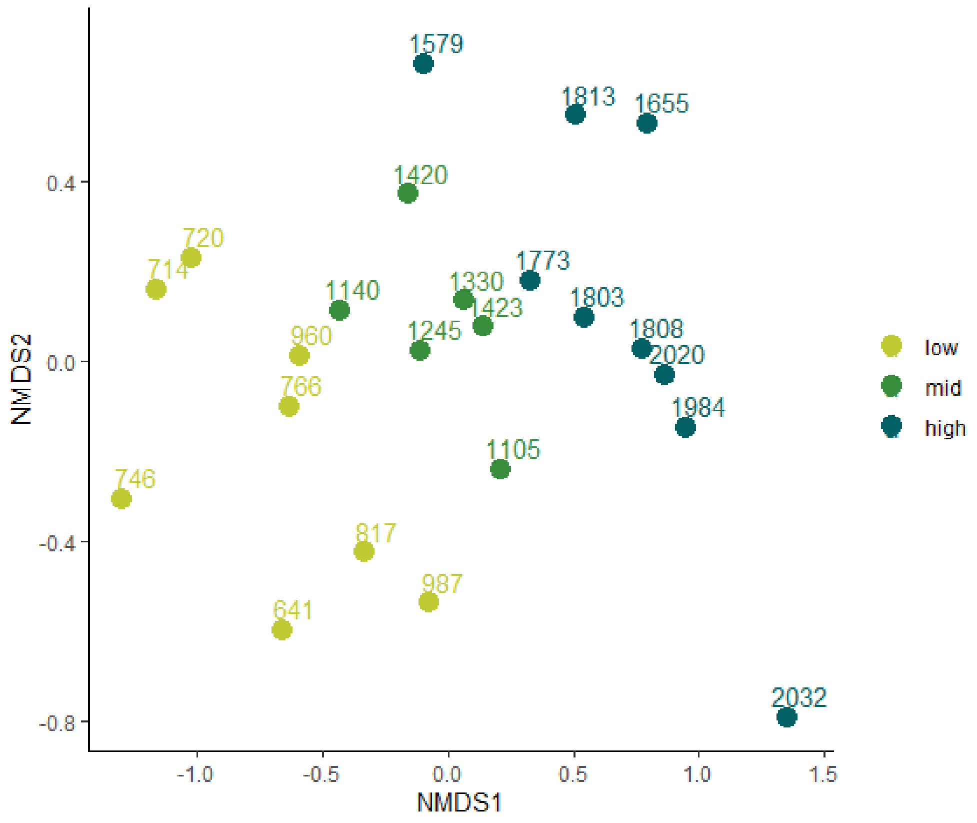

Figure S8. Plant community composition along the elevational gradient. *Each dot represents one site. Color code indicates elevational belt (low: 641-1017 m.a.s.l. mid 1018- 1448 m.a.s.l.; high 1449- 2200 m.a.s.l). Each site is labelled with respective elevation (m.a.s.l))*

Section S12: Test statistic to reveal rel. abundance change of 15 most important ASVs comparing translocated workers in lowland and mountain region.

Table S3. Abundance-based change of the 15 most important ASVs in the classification of samples from the dry and warm and the cold and wet region. *Presented are ASV IDs and Kruskal-Wallis results (Chi-square, p-value and mean difference) for respective species. Change indicates the change direction (+: increase in dry and warm; -: decrease in dry and warm; no: no change)*

Species	ASV.ID	chi-square	p-value	diffmean	change
B. terrestris	ASV.1165_Lactobacillaceae.sp	26.702	**<0.001**	0.020	+
B. terrestris	ASV.9_Snodgrassella	5.517	**0.019**	-0.058	-
B. terrestris	ASV.3_Apibacter	4.435	**0.035**	0.051	+
B. terrestris	ASV.404_Gammaproteobacteria.sp	1.756	0.185	0.076	no
B. terrestris	ASV.6_Acinetobacter	1.361	0.243	0.010	no
B. terrestris	ASV.56_Fructobacillus	11.278	**<0.001**	-0.010	-
B. terrestris	ASV.199_Saccharibacter	11.278	**<0.001**	-0.028	-
B. terrestris	ASV.1635_Saccharibacter	5.866	**0.015**	-0.030	-
B. terrestris	ASV.2_Acinetobacter	3.665	0.056	0.030	no
B. terrestris	ASV.12_Acinetobacter	4.848	**0.028**	0.011	+
B. terrestris	ASV.31_Gilliamella	1.154	0.283	0.002	no
B. terrestris	ASV.21_Acinetobacter	3.816	0.051	0.002	no
B. terrestris	ASV.1388_Bombilactobacillus	4.382	**0.036**	0.012	+
B. terrestris	ASV.2493_Bifidobacterium	2.816	0.093	0.001	no
B. terrestris	ASV.1172_Saccharibacter	2.235	0.135	-0.001	no
B. lucorum	ASV.1165_Lactobacillaceae.sp	17.416	**<0.001**	0.013	+
B. lucorum	ASV.1635_Saccharibacter	6.194	**0.013**	0.020	+
B. lucorum	ASV.1388_Bombilactobacillus	7.926	**0.005**	0.017	+
B. lucorum	ASV.483_Lactobacillus	7.926	**0.005**	0.016	+
B. lucorum	ASV.1_Gilliamella	5.472	**0.019**	-0.087	-
B. lucorum	ASV.6_Acinetobacter	3.632	0.057	0.006	no
B. lucorum	ASV.12_Acinetobacter	4.538	**0.033**	0.018	+
B. lucorum	ASV.33_Proteobacteria.sp	4.536	**0.033**	-0.009	-
B. lucorum	ASV.9_Snodgrassella	0.030	0.862	-0.051	no
B. lucorum	ASV.868_Apilactobacillus	3.799	0.051	-0.007	no
B. lucorum	ASV.1188_Secundilactobacillus	4.931	**0.026**	0.016	+
B. lucorum	ASV.489_Lactobacillus	4.538	**0.033**	0.007	+
B. lucorum	ASV.161_Proteobacteria.sp	3.799	0.051	-0.003	no
B. lucorum	ASV.92_Gilliamella	2.175	0.140	-0.001	no
B. lucorum	ASV.404_Gammaproteobacteria.sp	2.209	0.137	0.120	no

References

Anderson, M. J. (2001). A new method for non-parametric multivariate analysis of variance. *Austral Ecol.* 26, 32–46. doi: 10.1111/j.1442-9993.2001.01070.pp.x

Brenzinger, K., Maihoff, F., Peters, M. K., Schimmer, L., Bischler, T., and Classen, A. (2022). Temperature and livestock grazing trigger transcriptome responses in bumblebees along an elevational gradient. *iScience* 25, 105175. doi: 10.1016/j.isci.2022.105175

Maihoff, F., Friess, N., Hoiss, B., Schmid-Egger, C., Kerner, J., Neumayer, J., et al. (2023). Smaller, more diverse and on the way to the top: Rapid community shifts of montane wild bees within an extraordinary hot decade. *Divers. Distrib.* 29, 272–288. doi: 10.1111/ddi.13658
